# Simple Conditional Spatial Query Mask Deformable Detection Transformer: A Detection Approach for Multi-Style Strokes of Chinese Characters

**DOI:** 10.3390/s24030931

**Published:** 2024-01-31

**Authors:** Tian Zhou, Wu Xie, Huimin Zhang, Yong Fan

**Affiliations:** 1 Guangxi Key Laboratory of Image and Graphic Intelligent Processing, Guilin University of Electronic Technology, Guilin 541004, China; guetzt163@163.com; 2 School of Computer Science and Information Security, Guilin University of Electronic Technology, Guilin 541004, China; 3 Key Laboratory of Education Blockchain and Intelligent Technology, Ministry of Education, Guangxi Normal University, Guilin 541004, China; 4 Guangxi Key Lab of Multi-Source Information Mining and Security, Guangxi Normal University, Guilin 541004, China; 5 School of Mechanical and Electrical Engineering, Guilin University of Electronic Technology, Guilin 541004, China; fanysmee@126.com

**Keywords:** object detection, Chinese character stroke, transformer, deformable DETR, SCSQ-MDD

## Abstract

In the Chinese character writing task performed by robotic arms, the stroke category and position information should be extracted through object detection. Detection algorithms based on predefined anchor frames have difficulty resolving the differences among the many different styles of Chinese character strokes. Deformable detection transformer (deformable DETR) algorithms without predefined anchor frames result in some invalid sampling points with no contribution to the feature update of the current reference point due to the random sampling of sampling points in the deformable attention module. These processes cause a reduction in the speed of the vector learning stroke features in the detection head. In view of this problem, a new detection method for multi-style strokes of Chinese characters, called the simple conditional spatial query mask deformable DETR (SCSQ-MDD), is proposed in this paper. Firstly, a mask prediction layer is jointly determined using the shallow feature map of the Chinese character image and the query vector of the transformer encoder, which is used to filter the points with actual contributions and resample the points without contributions to address the randomness of the correlation calculation among the reference points. Secondly, by separating the content query and spatial query of the transformer decoder, the dependence of the prediction task on the content embedding is relaxed. Finally, the detection model without predefined anchor frames based on the SCSQ-MDD is constructed. Experiments are conducted using a multi-style Chinese character stroke dataset to evaluate the performance of the SCSQ-MDD. The mean average precision (mAP) value is improved by 3.8% and the mean average recall (mAR) value is improved by 1.1% compared with the deformable DETR in the testing stage, illustrating the effectiveness of the proposed method.

## 1. Introduction

With the rapid integration of artificial intelligence into the mechanical industry, the use of industrial robotic arms has increased [1,2,3]. However, at present, there are few robotic arms that can write good Chinese calligraphy characters on a flat surface. On the one hand, this is because the robotic arm does not have the same precise control of each stroke trajectory as the human arm. On the other hand, it is because the extent of processing of Chinese characters is not fine enough. Currently, there is no appropriate algorithm that can be used to perfectly predict the trajectory point of each stroke in every Chinese character, so the input parameters of the robotic arm cannot enable it to make a complete trajectory movement. The three elements of Chinese images include stroke, position, and sequence, i.e., each stroke of a Chinese character, the corresponding position of each stroke in the image, and the sequence of each stroke. Chao et al. [4] proposed the use of a corner point detection technique to decompose Chinese characters into a set of strokes, subsequently using the operator’s gestures to recognize the decomposed strokes as the robot’s writing trajectory to complete the robot’s Chinese character writing task. Wang et al. [5] proposed the use of a full convolutional network to extract the stroke skeleton and intersection region and track the whole stroke extraction process based on the pixels in the non-intersection region, finally using the tree search method to match the candidate strokes with the standard strokes to obtain the correct strokes. Although these methods have been successfully used to extract the strokes of Chinese characters and obtain the trajectory of the robot arm by processing the strokes, they are based on manual predefined rules to split the strokes, making them not truly unsupervised stroke extraction methods. Each stroke of a Chinese character and the specific position of each stroke are acquired in our work using object detection techniques in the field of image recognition.

Most previous object detection approaches aimed at improving the generation of proposal boxes and optimizing the filtering of proposal boxes by generating a series of sample candidate boxes using two-stage methods to classify the samples with convolutional neural networks (CNNs). These methods focused on improving detection accuracy and positioning precision, but the models’ detection speeds slowed down due to the use of two-stage detection methods [6,7,8,9,10,11] to map the candidate boxes to the corresponding area of the feature maps after generating them. The generation of sample candidate boxes was removed, and the problem of the localization of target boxes was directly transformed into a regression prediction problem using one-stage methods. These methods focused on addressing the problem of slow detection speeds, but they are inferior to the two-stage methods as far as detection accuracy and positioning precision are concerned. Redmon et al. [12] proposed the You Only Look Once (YOLO) model to predict two bounding boxes and multiple category scores for each grid cell on the feature map and continuously update the values of the bounding boxes and category scores through a loss function. The average precision (AP) value on the Pascal VOC 2007 test dataset reached 63.4%, yet YOLO has the limitation of poor detection on small targets in groups. Therefore, Redmon et al. [13] proposed YOLOv3, which integrated low-level and high-level features by adding a feature pyramid network (FPN) structure and predicted three different feature layers, enabling the model to detect objects of different scales. The AP value on the COCO dataset reached 33.0%, but YOLOv3 has limitations such as imbalanced positive and negative samples and sensitivity to grid boundary values. Bochkovskiy et al. [14] proposed YOLOv4 based on this problem to redesign the sample matching criterion to reduce the impact of the positive and negative sample imbalance. YOLOv4 eliminated the grid sensitivity problem through the design of an activation function, and the AP value on the COCO dataset reached 41.2%. But YOLOv4 still has the problem of poor matching of manually designed anchor boxes on different tasks. Due to the excessive number of candidate boxes generated during the prediction process of one-stage methods [15,16,17,18,19,20], non-maximum suppression (NMS) processes are required to filter out a large number of candidate boxes, which not only reduces the inference speed but also fails to achieve truly end-to-end prediction, as shown in [21,22], due to the incorporation of a supervisory mechanism.

With transformer methods achieving good results in the natural language processing (NLP) field, researchers have also attempted to introduce transformers into the computer vision (CV) field. Bello et al. [23] proposed adopting self-attention as an alternative approach to convolutional neural networks for discriminating visual tasks to address the limitation of convolutional blocks only being calculated with local neighborhoods, resulting in a lack of global information. Because the traditional CNN model structure can only be utilized to model local information, it is difficult to model long-period information. The attention model has a strong periodic modeling ability, so self-attention can make up for the deficiency of CNNs in ultra-long-period modeling. To compensate for the lack of spatial position information in the transformer, Shaw et al. [24] proposed combining relative position encoding and a self-attention mechanism for modeling position information in images. Furthermore, Ramachandran et al. [25] proposed the use of only attention and relative position encoding instead of the convolutional module in a deep residual network (Resnet) to achieve an image model with full attention. Dosovitskiy et al. [26] presented a vision transformer (ViT) and converted images into token sequences that can be received by the transformer encoder through patch embedding, allowing the operation processes of multi-head self-attention to be performed on the image feature map. Meanwhile, Carion et al. [27] proposed a detection transformer (DETR) that flattens the feature map obtained from the images through the backbone. The feature map is converted into a token sequence, allowing it to be processed by the transformer encoder. The memory vector obtained from the encoder and the 100 object queries obtained from the decoder, after self-attention updating, are subjected to a cross-attention operation. Finally, the classification and regression values of the 100 queries are predicted. Using this method, many manual design components, such as the generation of sample candidate boxes and NMS processing, are effectively eliminated. Wu et al. [28] proposed improved relative position encoding (iRPE) and combined relative position encoding and absolute position encoding in a DETR, resulting in a 1.3% increase in the AP value compared with only using absolute position encoding. Chen et al. [29] proposed a group DETR by employing multiple groups of object queries and performing one-to-one label assignments for each group to support grouped one-to-many assignments, addressing the limitations of DETR, which relies on one-to-one assignments and lacks the ability to utilize multiple positive object queries. Bar et al. [30] presented an unsupervised pretraining method with region priors for object detection, known as DETReg, to pretrain the entire DETR detection network by extracting the proposal box and predicting the self-supervised image coding of regions through an object localization task and an object embedding task during pretraining. The corresponding feature embedding with the self-supervised image coding embedding is aligned to achieve the goal of pretraining the whole DETR detection network.

However, since every point needs to be calculated with all other points in the attention computing module of the DETR, the convergence is slow, and the image resolution is limited. Li et al. [31] attributed the slow convergence of the DETR to the discreteness of the Hungarian matching algorithm and the randomness of model training, leading to ground-truth (GT) box matching becoming a dynamic and unstable process. The DN-DETR (DeNoising DETR) was proposed to reconstruct the GT box by feeding the GT box with noise into the transformer decoder and training the model. Since this process does not require Hungarian matching, the difficulty of binary graph matching is effectively reduced, and the convergence speed is accelerated. Zhang et al. [32] attributed the slow convergence speed of the DETR to the complexity of matching object queries with target object features in different feature embedding spaces. They proposed the SAM-DETR (semantic-aligned matching DETR), wherein object queries are projected into the same embedding space as encoded image features, and then semantic alignment matching is performed, thereby improving detection accuracy and speeding up convergence. Gao et al. [33] argued that the reason for the slow convergence of the DETR is that the object query vector of the DETR needs to interact with the global features of the image so the decoder needs a long training time for the object query to accurately locate the object. The SMCA (Spatially Modulated Co-Attention) mechanism was proposed to improve the convergence speed of the DETR by introducing the Gaussian distribution model of objects into the common attention mechanism and adjusting the search range of each object query vector in the common attention mechanism within a certain distance near the object center.

Kitaev et al. [34] analyzed the traditional transformer and emphasized that the distribution of long sequences is almost always sparse, which indicates that a feature point in a sequence is usually highly correlated with only a few other points. Therefore, only the connections between a subset of points and the current point need to be focused on during the computation of the attention module, which avoids the need for correlation calculations with all points when calculating attention in the transformer, thereby reducing the overall model computation. Zhu et al. [35] proposed the deformable DETR by incorporating deformable attention into the DETR, which requires calculating the connections of each sampling point and its surrounding key points, thereby addressing the problem of the excessive attention computation of DETR. Meanwhile, the multiscale feature map concatenating is used in deformable DETR to solve the problem of slow accuracy in detecting small objects in DETR. Experiments showed that the deformable DETR improved convergence speed and accuracy compared to the ordinary DETR; however, the deformable attention module should focus more on key sampling points with important features when sampling key points is not considered in the deformable DETR. Meng et al. [36] proposed the conditional DETR and also analyzed the reason for the slow convergence speed of the DETR. They found that cross-attention highly relies on content embedding to locate the position of the prediction box, so the demand for high-quality content embedding increases along with the training difficulty. Therefore, the decoupling of the content query and spatial query was proposed, and a learnable conditional spatial query module was introduced to enable the model to learn conditional spatial queries from the decoder embedding. This allows each cross-attention head to focus on different areas, narrowing the spatial range for object classification and prediction box regression in different regional positions.

**Figure 1:**
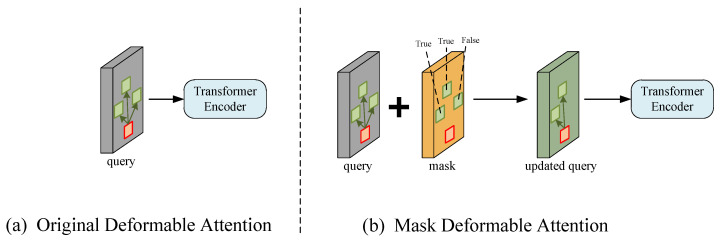
(**a**) The simple process of the deformable attention used in existing methods. (**b**) The simple process of the proposed deformable attention with a mask mechanism. The proposed mask mechanism determines whether a sampling point has a contribution value to the current reference figure point by predicting the mask layer and discards the sampling points that do not have a contribution value. The gray block represents the query vector, while the yellow block represents the mask vector corresponding to the query vector, and the green block represents query vector after filtering and resampling. The red box represents the current reference point and the green boxes represent the random sampling points.

The goal of the method proposed in this paper is to discard all hyperparameters associated with the anchor frame, enabling the use of the deformable DETR without a predefined anchor frame. The sampling points that do not contribute features during the deformable attention module sampling are resampled so that these points provide feature contributions to the reference points. It is found through experiments that setting the offset of certain sampling points to 0, i.e., discarding some sampling points, leads to a small performance improvement. The reason for this phenomenon is that some points in the random sampling process are repeatedly sampled. When these duplicate sampling points are removed, the computational efficiency of the model is superior to the model with duplicate sampling points, making it easier to converge. Our proposed mask deformable DETR is an improved end-to-end stroke detection method via a deformable DETR with a sampling region prediction mechanism. Figure 1 shows the difference between the deformable attention with the addition of the mask mechanism and the original deformable attention. A mask mechanism is introduced in our method to predict which sampling points are the likely regions of interest for the current reference point and to recalculate and adjust the sampling points in non-important areas. By removing the attention calculation for invalid sampling points, the computational efficiency of the deformable attention module is increased. The convergence speed of the model is accelerated and preferable performance is achieved in a short period of time.

The most primitive feature map extracted from the backbone and the updated query vector of each encoder layer are jointly utilized to sample, concatenate, and fuse by the deformable DETR based on the mask mechanism. This is done to predict which sampling points in the query vector are candidate points with contribution values for the current reference point. Resampling is used to assign values to candidate points without contribution. Moreover, the mask prediction layer can be simply embedded into the encoder layer of the transformer without the need to modify complex logical structures. Excellent performance improvements are achieved while reducing computational costs. The main contributions of this study can be summarized as follows:

(1) A multiscale deformable attention module based on a mask mechanism is proposed to improve computational efficiency and speed up the convergence of the model by predicting the key sampling points around each reference point in the query vector. In addition, the points that contribute features to the current reference point are filtered out, whereas points that do not contribute features are resampled.

(2) A simple conditional spatial query structure is introduced. By processing the content query vector and the spatial query vector and performing simple linear fusion, the separation of the content query and spatial query is accomplished without introducing additional parametric quantities. The model can be used to focus on not only the content embedding but also the spatial embedding when performing cross-attention calculations. The dependence of the prediction task on content embedding is relaxed, and the training process is simplified.

(3) A splitting feedforward network (SFN) structure is proposed to perform split and cross-fusion calculations on the output vectors from the transformer decoder. To the best of our knowledge, this is the first work to apply the simple conditional spatial query mask deformable DETR (SCSQ-MMD) with an SFN module in the field of deformable DETR. Then, classification and regression predictions are performed in the SFN to enhance the focus on different features for classification and regression tasks.

In short, for the Chinese character writing task performed by robotic arms, an accurate and efficient algorithm is needed to support the detection of Chinese character strokes, especially the implementation of a complete end-to-end stroke detection method. In this paper, the deformable DETR model is improved by enhancing stroke detection accuracy through the above three novel contributions. Experimental results show that the stroke detection method proposed in this paper is superior to the traditional deformable DETR detection method, which can assist robotic arms in completing the Chinese character writing process.

## 2. Related Works

The network model in this study is improved using the deformable DETR and further extended through the use of the conditional DETR. The deformable DETR is introduced briefly in Section 2.1, and the idea of the conditional DETR is introduced in Section 2.2.

### 2.1. Deformable DETR and Multiscale Deformable Attention Mechanism

The deformable DETR [35] incorporates a multiscale deformable attention mechanism based on the DETR. First, a query vector is obtained by concatenating the input feature maps of multiple scales, which is fed into the encoder. Each reference point in this vector directly predicts *k* random offsets around the current point. Second, these *k* offsets are mapped to the query vector for sampling, and then the final value obtained by linearly interpolating the features of these *k* points is used to update the features of the current reference point.

The self-attention mechanism obtains the weight coefficients for each value by calculating the correlation between each query and the other keys in this vector. The weight coefficients and the corresponding value are then weighted and summed to obtain the final attention value. In this way, the connections between each point in the vector and the other points can be obtained by the attention module, and the interdependent features in the vector can be captured.

In the multi-head attention mechanism, the self-attention module is used to calculate for each head, without sharing parameters between each head. The final result is obtained by concatenating and fusing the results of the self-attention computed by multiple heads. The formula for calculating the multi-head attention mechanism is as follows:

(1) $$\begin{array}{c} \mathrm{MultiHeadAttn}\left(z_{q},x\right)=\sum_{m=1}^{M}W_{m}\left[\sum_{k\in \Omega }A_{mqk}\cdot W_{m}^{\prime }x_{k}\right] \end{array}$$

where *q* indexes a query element with the representation feature $z_{q}$, *k* indexes a key element with the representation feature $x_{k}$, and *m* indexes the attention head. $W_{m}$ and $W_{m}^{\prime }$ are the trainable weights. $A_{mqk}$ represents the attention weights of the *k*-th point in the *m*-th attention head.

The multiscale deformable attention mechanism is based on the common multi-head attention mechanism and adds sampling offsets to each attention head of each scale. The mechanism involves sampling the key of the local position in the global position for each query to obtain the value of the corresponding local position. Finally, the local attention weight and the local value are calculated to reduce the computation of attention, thereby accelerating the convergence speed of the model. The formula is as follows:

(2) $$\begin{array}{c} \mathrm{MSDeformAttn}\left(z_{q},p_{q},x\right)=\sum_{m=1}^{M}W_{m}\left[\sum_{k=1}^{K}A_{mqk}\cdot W_{m}^{\prime }x\left(p_{q}+\Delta p_{mqk}\right)\right] \end{array}$$

where *m* indexes the attention head, *k* indexes the sampled key, *K* is the total number of sampled keys (k≪HW), and $\Delta p_{mqk}$ and $A_{mqk}$ are the sampling offsets and attention weights of the *k*-th sampling point in the *m*-th attention head, respectively.

### 2.2. Conditional DETR and Conditional Spatial Query Module

The reasons for the slow convergence of the DETR were analyzed in [36]. The spatial query only utilizes the common attention weight information and not the specific image information. The content query has to match both the spatial keys and content keys, meaning there is no way for it to learn good features in a short time. The attention weights for the cross-attention mechanism in the DETR are calculated based on the dot product between the query and the key. The formula is as follows:

(3) $$\begin{array}{c} {\left(c_{q}+p_{q}\right)}^{T}\cdot \left(c_{k}+p_{k}\right)=c_{q}^{T}c_{k}+c_{q}^{T}p_{k}+p_{q}^{T}c_{k}+p_{q}^{T}p_{k} \end{array}$$

where $c_{q}$ is the content query, $c_{k}$ is the content key, $p_{q}$ is the spatial query, and $p_{k}$ is the spatial key.

By forcing the separation of content queries and spatial queries in the conditional cross-attention mechanism, content queries and spatial queries can focus on content attention weights and spatial attention weights, respectively. The content attention weights and spatial attention weights are derived from the content dot product and the spatial dot product, respectively. The formula is as follows:

(4) $$c_{q}^{T}c_{k}+p_{q}^{T}p_{k}$$

A learnable conditional spatial query strategy is introduced in the conditional DETR to learn the conditional spatial query vectors from decoder embeddings for decoding multi-head cross-attention. Specifically, the conditional space query $p_{q}$ is obtained by dot-producting the sine and cosine encoding results $p_{s}$ of the reference point *s* with the linear mapping result T of the embedding *f* output by the decoder at the previous layer.

(5) $$p_{q}=T\cdot p_{s}=\mathrm{FFN}\left(f\right)\cdot (\mathrm{sinusoidal}(\mathrm{sigmoid}(s)))$$

The input query vector of the cross-attention module is obtained by concatenating the conditional spatial query $p_{q}$ and the encoding $c_{q}$ obtained by the self-attention module.

In this approach, the high dependence on content embedding is reduced by separating the spatial queries and content queries, allowing them to focus on spatial attention weights and content attention weights, respectively.

## 3. Methods

### 3.1. Mask Deformable Attention in the Multiscale Deformable Attention Module

**Figure 2:**
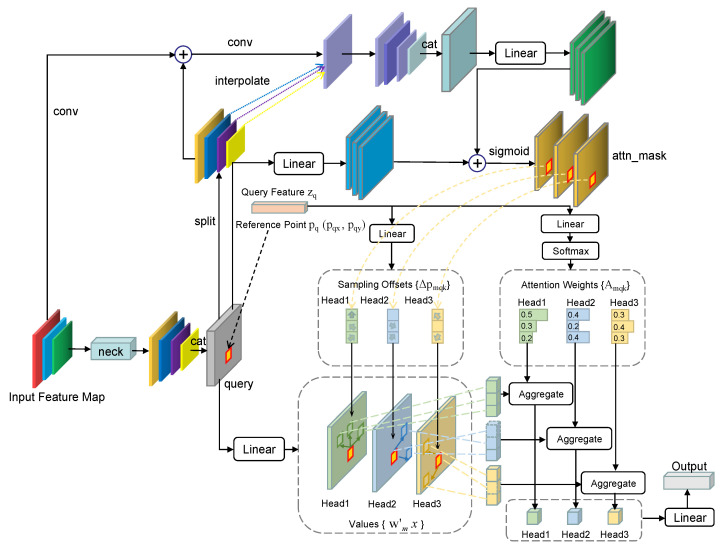
The proposed deformable attention module based on the mask mechanism. The purpose of the proposed mask mechanism is to generate a mask prediction layer that predicts which random sampling points have not contributed to the current reference point and discards these points. The random sampling process is accomplished by adding random integer offsets to the current reference point to obtain the exact position of the sampling points. Blocks of different colors represent different vectors, and blocks of different sizes represent different sized feature maps. The dotted lines represent the correspondences between the different blocks.

The core of the deformable DETR model with the mask attention mechanism is the multiscale deformable attention module with the mask mechanism. Figure 2 shows the complete structure of this deformable attention module. The feature map obtained by the backbone contains the most accurate foreground location information from the original image, reflecting the location of the object and the size of the region in the original image. In order to use the mask to accurately predict whether each sampling point in the query vector contributes features to other reference points, the foreground position information from the feature map of the original image is needed. The same operation used in the original deformable DETR is adopted to concatenate the multiscale feature map into a query vector, which is then fed into the multiscale deformable attention module for computation. The generation process of the mask prediction layer and the filtering process of the sampling points are as follows: (1) The upper-layer feature map with the least missing information undergoes convolution to obtain object region position information, which is then fused with the features processed by the channel mapper. (2) The feature maps from several other levels are sampled to obtain information from the feature map of each level corresponding to the object region position. (3) The position information of object regions from multiple levels is fused and concatenated to generate a mask prediction layer. This mask layer is used to predict whether *k* key sampling points of each reference point $z_{q}$, obtained from the query vector through linear mapping, have contributed features to the current reference point. *k* is the number of sampled points. Since the query vector updated by each encoder layer contains the latest information of the current reference point, the mask prediction layer needs to be updated by each encoder layer to ensure that the mask always learns the crucial predicted features.

The deformable multi-head attention module with the mask mechanism is used not only to sample the key of the local position in the global position for each query vector but also to filter the local sampling points according to the value predicted by the mask. Sampling points without feature contributions are resampled to obtain new keys, and the updated local attention weights are then multiplied by local values. This process reduces the computation in the attention module. Sampling points without feature contributions are filtered out to avoid useless computation on points without contributions. The convergence speed of the model is further accelerated, and higher detection accuracy is achieved in a short period of time. The calculation formula for the deformable attention module with the mask mechanism is as follows:

(6) $$\begin{array}{c} \mathrm{MMSDeformAttn}\left(z_{q},p_{q},x\right)=\sum_{m=1}^{M}W_{m}\left[\sum_{k=1}^{K}A_{mqk}\cdot W_{m}^{\prime }x\left(p_{q}+mask_{mqk}\cdot \mathrm{func}\left(\Delta p_{mqk}\right)\right)\right] \end{array}$$

where *m* indexes the attention head, *k* indexes the sampled keys, *K* is the total number of sampled keys (k≪HW), and $\Delta p_{mqk}$ and $A_{mqk}$ are the sampling offsets and attention weights of the *k*-th sampling point in the *m*-th attention head, respectively. $mask_{mqk}$ is the mask value corresponding to the *k*-th sampling point in the *m*-th attention head ($mask_{mqk}\in$ [True, False]), which determines whether the current point needs to be resampled for calculation. The symbol func is the resampling function with two strategies: non-sampling and value decay.

The difference between the proposed mask deformable attention mechanism and the attention weights in the original DETR is that although the original DETR can adjust the importance of different contributing points through attention weights, it computes the query vectors of each point with the key vectors of all other points. Although the importance of different contributing points can be adjusted using this approach, there is no way to reduce the number of computations. Even when the attention weight of a point is 0, the query, key, and value of that point still participate in the self-attention calculation process. In the mask deformable attention mechanism, each query vector is computed only with the key and value of the random sampling set of points around it. A resampling strategy is adopted in this mechanism to address the problem of non-contributing sampling points caused by random sampling, which replaces non-contributing points with other points close to the current reference point. This makes the model carry out a more effective attention computation process, thereby accelerating its convergence speed.

**Figure 3:**
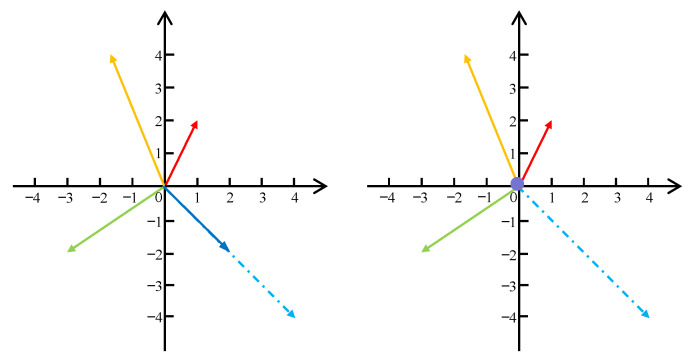
The resampling strategy of sampling points in the deformable attention module based on the mask mechanism. The offset in the same direction for a sample point that does not contribute to the current reference point is decreased, and this invalid reference point is replaced with another sample point in that direction that is closer to the reference point. Arrows of different colors represent different sampling points, and the coordinates where the arrows are located represent the offsets from the current reference point. Dashed arrows represent the original sampling point, solid arrows in that direction represent a reassignment of the sampling offset, and the dot represents discarding the current offset.

The resampling strategy of the F function is shown in Figure 3, with the direction-invariant and value-nonlinear decay strategy on the left, and the non-sampling strategy on the right. The non-sampling strategy sets the sampling offset value of the *k*-th sampling point in the *m*-th attention head to 0, indicating that the point corresponding to the predicted offset does not have a contribution value and is directly discarded. The strategy of direction-invariant and value-nonlinear decay is formulated as follows:

(7) $$\begin{array}{c} F\left(\Delta p_{x},\Delta p_{y}\right)=\left(\frac{\Delta p_{x}}{\Delta p_{x}}\times \sqrt{\left|\Delta p_{x}\right|},\frac{\Delta p_{y}}{\Delta p_{y}}\times \sqrt{\left|\Delta p_{y}\right|}\right) \end{array}$$

where $\Delta p_{x}$ and $\Delta p_{y}$ are the predicted *x* offset and *y* offset, respectively. $\left|\Delta p_{x}\right|$ and $\left|\Delta p_{y}\right|$ represent the absolute values of $\Delta p_{x}$ and $\Delta p_{y}$ respectively. When a certain point is predicted to be a non-critical point, the points of all regions with increasing values in this direction are also non-critical points. So, the offset needs to be updated using a constant direction and nonlinear decay of value. Specifically, the random sampling process involves adding an integer-valued random offset to the current reference point to obtain the specific position of the sampling point. If a point at a certain location is predicted to be a non-contributing point by the mask layer, the value of the random offset nonlinearly shrinks in the current direction to become closer to the position of the reference point using the linear decay strategy in Equation (7). With this strategy, non-contributing points can be replaced with other points around the reference point, thus leading to more efficient attention computations by the model.

### 3.2. The Simple Conditional Spatial Query Strategy

The conditional DETR can be used to speed up the convergence of the model and improve detection accuracy. But, owing to the multiscale concatenating mode used in the deformable DETR, the size of the query vector of the input encoder is too long. The direct incorporation of the conditional spatial query module not only significantly increases the number of computations but also compromises the generality of the deformable attention module. Specifically, too many linear mappings are used in the conditional spatial query module in the original conditional DETR, which increases the number of computations and contrasts with the original intention of the design of this study—to reduce the number of computations. Moreover, the implementation of the original conditional DETR conflicts with that of the deformable DETR, which means the conditional spatial query module of the conditional DETR cannot be used directly in the proposed mask deformable DETR. In this study, the experiments prove that the detection performance of the deformable DETR is instead reduced by using the complex sub-module structure.

Therefore, the separation operation of the content queries and spatial queries in the self-attention module is discarded through the proposed simple conditional spatial query (SCSQ) strategy in this study. The strategy of the original conditional spatial query in the cross-attention module is simplified. In order to reduce the number of additional parameters introduced and preserve the generality of the deformable attention module, some of the linear mapping processes are omitted in the decoder in the simple conditional spatial query. After the query vector and the conditional spatial query vector are concatenated, feature fusion is performed through a linear layer to restore the dimensionality of the feature vector to its original length. These modification processes allow the application of the deformable DETR using the mask mechanism to the simple conditional spatial query module in the decoder. The separation of content queries and spatial queries is achieved by introducing a small number of additional parameters. Therefore, the model can focus on content embeddings and spatial embeddings separately when cross-attention computation is performed. Thus, the dependence of the prediction task on the content embeddings is relaxed, and the training processes are simplified.

The conditional cross-attention is formed by connecting the content query, the output of the self-attention in the decoder, and the spatial query. The keys consist of the content keys and spatial keys. The formula for calculating the conditional cross-attention is as follows:

(8) $$\begin{array}{cc} \mathrm{CondCrossAttn}(k,k_{pos},q,q_{pos},v)= & \mathrm{proj}\left(\left[\mathrm{SelfAttn}\left(q,k,v,q_{pos}\right),\mathrm{CondSpatial}\left(q,q_{pos}\right)\right]\right)\cdot \mathrm{proj}{\left(\left[k,k_{pos}\right]\right)}^{T}\cdot v \end{array}$$

where *k* and *v* are the memory vectors output by the encoder, $k_{pos}$ is the 2D spatial position information input by the encoder, *q* is the query input by the decoder, $q_{pos}$ is the 2D spatial position information corresponding to the query input of the decoder, and [,] is the concatenation operation. The proj symbol is the simple linear mapping function. The SelfAttn function is a common self-attention mechanism calculation process. The formula for SelfAttn is as follows:

(9) $$\begin{array}{c} \mathrm{SelfAttn}\left(q,k,v,q_{pos}\right)=\left(q+q_{pos}\right)\cdot \left(k+k_{pos}\right)\cdot v \end{array}$$

where $q_{pos}$ is the spatial embedding of the *q* vector, as shown in Formula (8). CondSpatial is the calculation process of the conditional spatial query, and the formula is as follows:

(10) $$\begin{array}{c} \mathrm{CondSpatial}\left(q,q_{pos}\right)=\mathrm{FFN}\left(q\right)\cdot P_{s}\left(q_{pos}\right) \end{array}$$

where FFN is the multiple linear mapping layers, and $P_{s}$ is the projection process of position encoding. The formula for $P_{s}$ is as follows:

(11) $$P_{s}\left(q_{pos}\right)=\mathrm{sinusoidal}\left(\mathrm{sigmoid}\left(q_{pos}\right)\right)$$

where sinusoidal represents a sine and cosine positional encoding function.

### 3.3. SFN Structure and Cross-Fused Module

**Figure 4:**
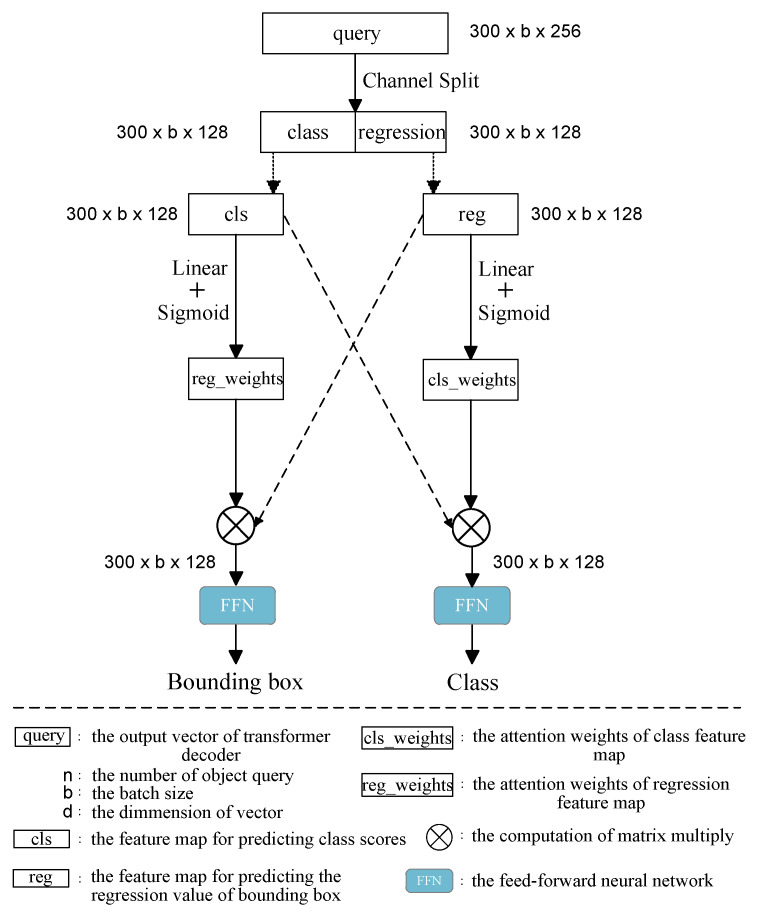
The structure of the proposed SFN module. The corresponding weight matrices are obtained from the vectors after linear and sigmoid computations. The weight matrices of the classification features are used as the weight coefficients of the regression task, and the weight matrices of the regression features are used as the weight coefficients of the classification task.

The traditional feedforward network (FFN) structure consists of a stack of multiple linear layers. The query vector is obtained through cross-attention computation using the memory obtained by the encoder after self-attention and the object queries of the decoder. This query vector is used to predict both the classification task and the regression task. Since the focuses of the classification task and regression task are different, the features they focus on should also differ. The classification task should focus more on stroke category information in the query vector, whereas the regression task should focus more on stroke position information. Therefore, a channel-splitting FFN structure is proposed in this study, called the splitting feedforward network (SFN) structure, which is shown in Figure 4. This structure initially splits the query vector output by the decoder into two different vectors, which are each used to predict different tasks. The problem caused by simple splitting is that the former and latter parts of features only focus on their own prediction tasks, resulting in the loss of correlations between the two parts. Considering that features important for classification may be potentially useful for the regression task, and features important for regression may be equally useful for the classification task, an alternative approach is considered. This approach involves cross-computing the former and latter parts of features, so a cross-fused module is proposed in this paper. This module facilitates the interaction between the corresponding weights of the features in the first part of the SFN and the features in the second part of the SFN. Similarly, it allows the corresponding weights of the features in the second part of the SFN to influence the first part of the SFN. In this way, both independence and correlation can be simultaneously emphasized by the former and latter parts of features. The resulting output features for the former part of the SFN are used for predicting the classification task, whereas the resulting output features for the latter part are used for predicting the regression task, thereby enhancing the independence of different prediction tasks. Specifically, the classification features are utilized to obtain a weight matrix through the linear layer, which is then applied to the regression task. The regression features are utilized to influence the classification task through a weight matrix obtained from the linear layer. In this way, the correlation between the classification task and the regression task can be strengthened. The *class* vector for the classification task and the *bounding box* vector for the regression task are obtained using the following equations:

(12) $${\mathrm{Vector}}_{\mathrm{cls}}=\mathrm{FFN}\left(\sigma \left(\mathrm{Linear}\left(\mathrm{reg}\right)\right)\cdot \mathrm{cls}\right)$$

(13) $${\mathrm{Vector}}_{\mathrm{bbox}}=\mathrm{FFN}\left(\sigma \left(\mathrm{Linear}\left(\mathrm{cls}\right)\right)\cdot \mathrm{reg}\right)$$

The difference between the SFN module proposed in this paper and the decoupled detector head in YOLO is that the decoupled detector head in YOLO ensures that the classification and regression tasks focus more on their features of interest by introducing classification and regression branches, allowing the detector head to converge faster and reducing latency while maintaining accuracy. However, the number of channels is reduced by slicing the channel of the query vector in the SFN module proposed in this paper to halve the computation of the detection head. At the same time, there is an intrinsic connection between the classification task and the regression task, e.g., the feature regions that are concerned with the classification features are also concerned with the regression task, and therefore cross-weights are used to strengthen the connection between the classification task and the regression task.

### 3.4. SCSQ-MDD Pipeline

**Figure 5:**
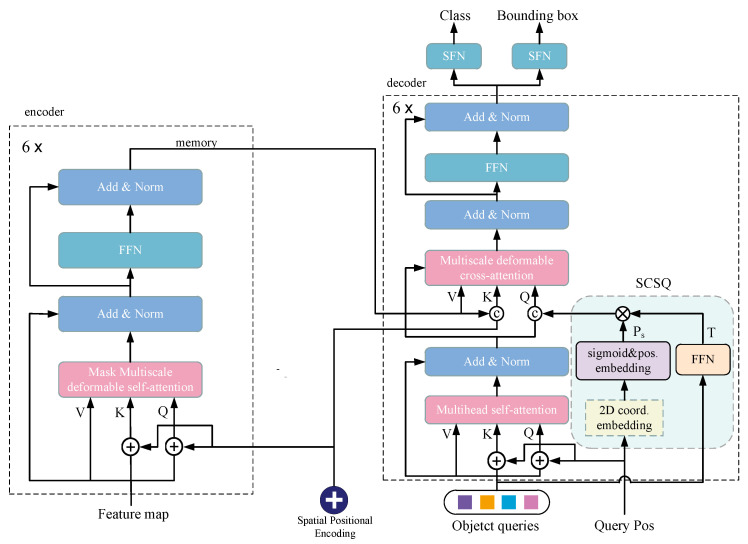
The overall network structure of the proposed SCSQ-MDD (simple conditional spatial query mask deformable DETR). The mask deformable attention mechanism proposed in this paper is incorporated into the improved multiscale deformable self-attention module. The purpose of the SCSQ is to obtain a spatial query vector, which is spliced and fused with the content query vector and used for cross-attention computations).

The overall structure of the SCSQ-MDD (simple conditional spatial query mask deformable DETR) is shown in Figure 5. The overall process of the model can be described as follows: (1) The input image is fed into the Resnet feature extraction network to obtain three feature maps of different scales, and the number of channels of feature maps of the three scales is then unified by the channel mapper, followed by a convolution operation to obtain the feature map of the fourth scale. (2) The feature maps of four scales are concatenated to obtain the feature vector (encoder embeddings), which contains image information from four different scales. The absolute position encoding of encoder embeddings is obtained using the sine and cosine position encoding methods. The query, key, value, unprocessed three-layer feature maps, and absolute position encoding obtained from encoder embeddings are input into the encoder. The mask layer is predicted in each encoder layer using the query and the unprocessed three-layer feature maps. In the deformable attention module, the mask layer specifies which sampling offsets need to be updated. The updated query vector is obtained in each encoder layer, and the model predicts a new mask layer based on the updated query vector, ensuring that the latest features can always be learned by the mask. The query, updated after six encoder layers, serves as a memory vector and undergoes cross-attention calculations with the object queries of the decoder. (3) By initializing the object queries and their corresponding positional encoding, the object queries, positional encoding, and memory vector obtained from the encoder can be input into the decoder. Initially, the calculations for the query positions and object queries are performed using a simple conditional spatial query to obtain the vectors (query embedding) of the conditional spatial query in each layer of the decoder. Then, calculations of the query, key, and value from the object queries are carried out with self-attentions to obtain the updated query vectors. The query vectors and the conditional spatial query vectors are concatenated and linearly fused to obtain new queries, which are vectors calculated and fused separately after isolating the content query and spatial query. At the same time, the memory vector serves as the key and value in the encoder used for cross-attention calculations with the new query, and the updated query is obtained through the deformable attention module. (4) After six decoder layer updates, the final query vector is obtained, which is then segmented and cross-fused. The first part of the features is used for predicting classification tasks, whereas the latter part is used for predicting regression tasks.

### 3.5. Chinese Stroke Detection Method Based on the SCSQ-MDD

**Figure 6:**
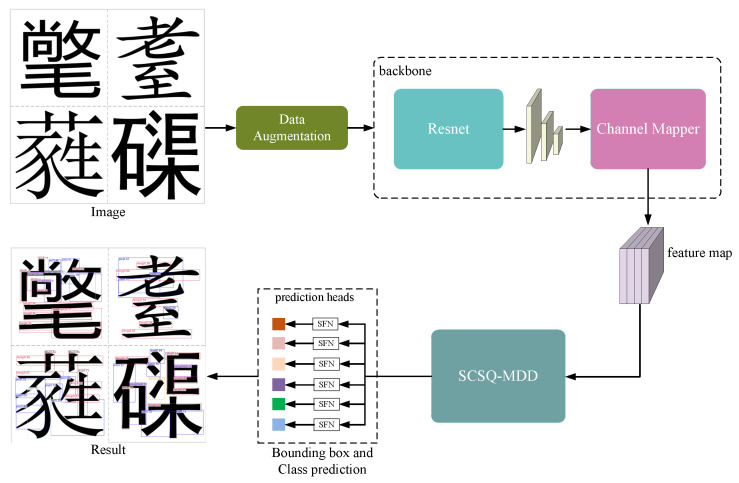
The overall flow of the Chinese character stroke detection network. Each Chinese character image consists of one style of stroke, and there are a total of 5 stroke styles of Chinese character images as input. The purpose of the backbone is to obtain the basic features of the image. The proposed SCSQ-MDD method is used to generate a high-level representation containing information about the location of the strokes and the category of the strokes of the Chinese characters, which is ultimately used in the head detector.

Figure 6 shows the overall flow of the Chinese character stroke detection method based on the SCSQ-MDD. The inputs are images of Chinese characters with five different stroke styles: “SimKai”, “SimHei”, “SimSun”, “MSYH”, and “Deng”. First, the sample data are processed using data augmentation methods, such as random flip, resize, and random crop, to improve the model’s detection generalization ability. Second, the sample data are fed into the Resnet network to extract features from both the Chinese character images and the Chinese character strokes. Third, since the original Chinese character images contain direct connections between strokes, the original three-layer feature maps extracted by Resnet and the current query vector are used to jointly predict the mask layer. Then, this mask layer is used to filter valid reference points in the query vector, discarding invalid reference points or resampling them as valid reference points using Equation (7). The attention is calculated only for the valid reference points using Equation (6), reducing the attention calculation process for the invalid reference points. This accelerates the learning of stroke feature information in the Chinese character image by the query vector. Fourth, the query vector of the decoder is obtained by performing simple conditional spatial query calculations using the object queries of the decoder and the 2D positional embedding vector. By separating the content query and the spatial query, the representation features of the detection boxes for the strokes can be learned faster by the query vector. Cross-attention calculations are performed between the memory vector updated by the encoder and the query vector of the decoder. The components of each query vector focus on the feature information of a stroke in the Chinese character image. The query vector obtained after six updates contains both the stroke feature information and the stroke position information. Finally, separate classification and regression predictions on query vectors are performed through the SFN module. The stroke detection results are visualized based on the category scores and the parameters of the regression detection box.

### 3.6. Application of the SCSQ-MDD to Robotic Arms

**Figure 7:**
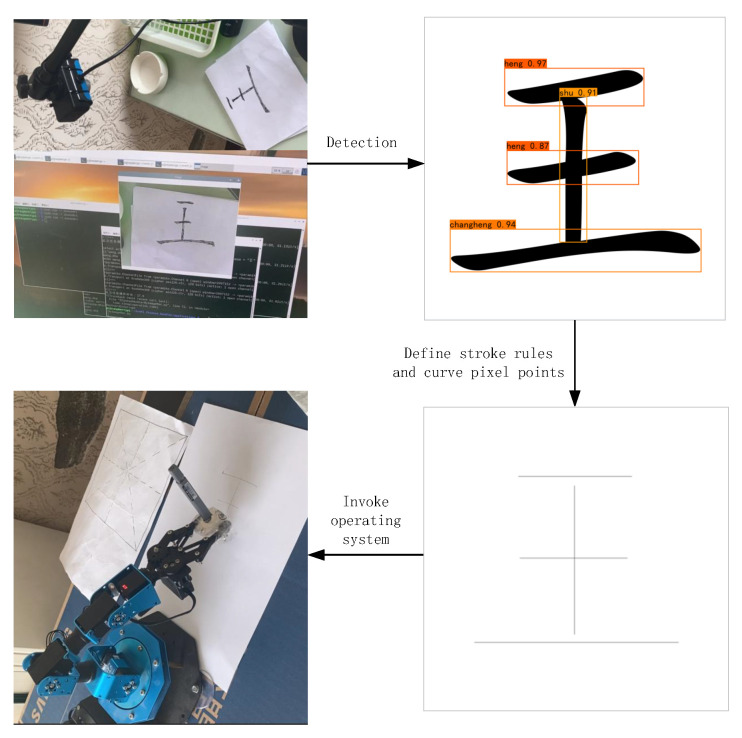
The process of applying the SCSQ-MDD method to robotic arms. A Chinese character consists figure of multiple strokes, and the position and category information of all the strokes are obtained by stroke detection.

The method proposed in this study can be applied to robotic arms for Chinese character writing tasks. Figure 7 shows the process of applying the SCSQ-MDD stroke detection method to robotic arms. First, the images of Chinese characters captured by the camera were recognized, and standard Chinese character images were generated based on the recognition results. Second, the SCSQ-MDD stroke detection method was used to detect all the strokes of the standard Chinese characters. Then, the reduction rules of the strokes were defined, and the pixel points of each stroke were reduced using the detected stroke categories and stroke positions. Finally, a set of pixel points was passed as a parameter to the listening program of the robotic arms, and the operating system of the robotic arms was used to complete the writing task of Chinese characters.

## 4. Experiments and Results

Since there is no publicly available Chinese character stroke dataset, the experimental data used in this paper were from a self-labeled standard Chinese character stroke dataset containing 1200 standard Chinese character images with five different styles and 52 stroke categories. Details of the 52 stroke categories are shown in Table 1. This dataset was divided into three sub-datasets for different tasks, where the training sets were used for model training, the validation sets were used to evaluate the detection metrics, and the test sets were used to test the model. The Pytorch framework was used to deploy the entire SCSQ-MDD model. In total, 800 standard Chinese character stroke images were used for model training, 200 standard Chinese character stroke images were used for model validation, and 200 standard Chinese character stroke images were utilized to test the model. The training platform used was a Quadro RTX 5000 graphics card with a batch size of 2. After 200 epochs of training, a total of more than 80,000 iterations were performed.

### 4.1. Implementation Details

The proposed SCSQ-MDD method used Resnet50/Resnet101 as the backbone to extract the basic features of Chinese character images. Six layers of transformer encoder layers were used on the transformer encoder side, and a mask multiscale deformable attention module with eight heads, embedding dimensions of 256, and four sample points was applied at each layer. Six layers of transformer decoder layers were used on the transformer decoder side, and a multi-head attention module with eight heads and embedding dimensions of 256 was applied at each layer. The classification loss used the FocalLoss function, with gamma set to 2.0 and alpha set to 0.25. The bounding box regression loss used the L1Loss function. The intersection over union (IOU) loss used the GIOULoss function. The Adam optimizer with a learning rate of 0.0002 was used.

### 4.2. Comparison between the SCSQ-MDD and the Deformable DETR

**Figure 8:**
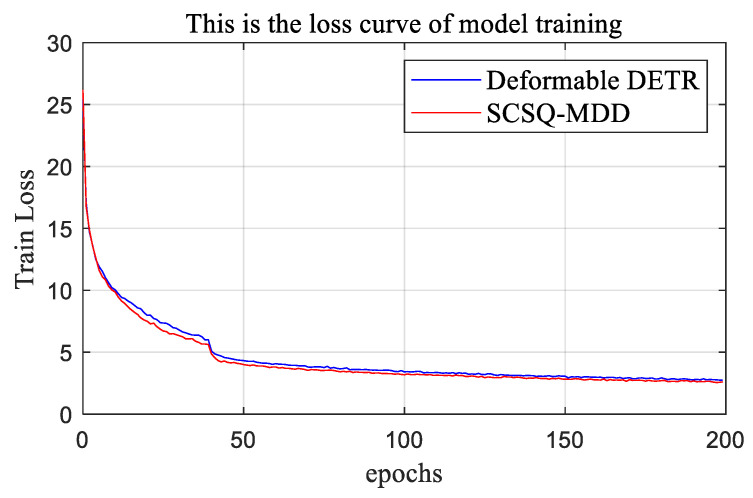
Trends in the loss function for the deformable DETR and the SCSQ-MDD during network training. The training loss of the proposed SCSQ-MDD decreased faster compared to the deformable DETR.

Figure 8 shows the trends in the loss function during network training for the deformable DETR and the SCSQ-MDD. The loss curves of both the deformable DETR and the SCSQ-MDD converged at the 50th epoch, with the loss curve of the SCSQ-MDD converging slightly faster than the loss curve of the ordinary deformable DETR.

**Figure 9:**
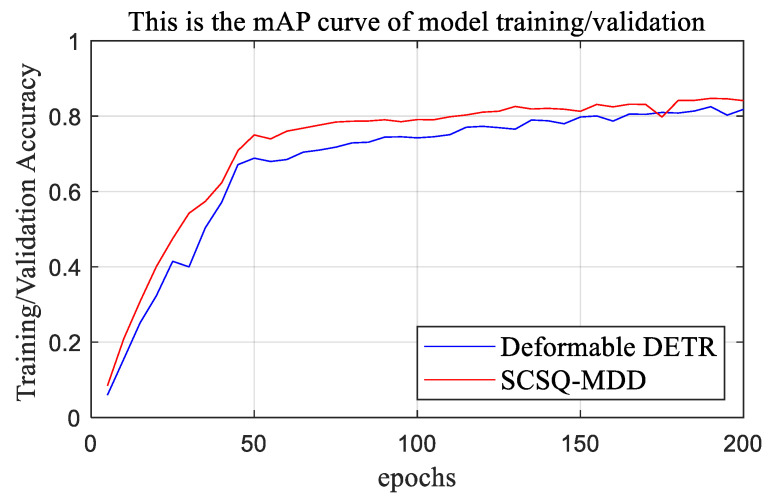
Trends in the accuracies of the deformable DETR and the SCSQ-MDD during the training/validation stage. The proposed SCSQ-MDD method performed significantly better compared to the deformable DETR method in terms of detection accuracy.

Figure 9 shows the trends in the accuracies of the deformable DETR and SCSQ-MDD networks during training/validation. It can be seen that the accuracy curve of the SCSQ-MDD lies slightly above the accuracy curve of the deformable DETR, demonstrating that the detection accuracy of the SCSQ-MDD method proposed in this paper is superior to that of the deformable DETR.

Table 2 shows the results of the comparison experiments using the deformable DETR and the SCSQ-MDD. It was found that our model resulted in substantial improvements in accuracy with only a slight increase in the number of parameters. Compared with the deformable DETR, the use of the mask deformable DETR increased the number of parameters by 1M, FLOPS by 41G, AP by 1.5%, AP50 by 2.0%, and AR by 0.8%. The use of the SCSQ-MDD increased the number of parameters by 6M, FLOPS by 41G, AP by 3.8%, AP50 by 3.1%, and AR by 1.1%. The FLOPS of the SCSQ-MDD exhibited almost no improvement compared to the mask deformable DETR, with increases in the AP of 2.3%, AP50 of 1.1%, and AR of 0.3%.

### 4.3. Comparison between the SCSQ Mask Deformable DETR and Mainstream Detection Methods

Table 3 shows the comparison results between the proposed SCSQ-MDD and mainstream detection methods. The AP of the method proposed in this paper was 88.1%, the AP50 was 95.6%, the AP75 was 95.5%, and the AR was 93.5%. Compared with Faster RCNN, the proposed method improved the AP by 9.6%, the AP50 by 0.5%, the AP75 by 1.6%, and the AR by 0.9%. Compared with ATSS, the proposed method improved the AP by 16.3%, the AP50 by 5.8%, the AP75 by 12.4%, and the AR by 14.5%. Compared with YOLOv5 with 1500 training epochs, the AP of our method with 500 epochs increased by 4% and the AP50 increased by 1.5%.

### 4.4. Ablation Study on Improved Mask Deformation Attention

Detailed ablation experiments were conducted for the SCSQ-MDD network structure design and the embedding of the mask deformable attention, SCSQ, and SFN modules. The performance of the network model was evaluated by comparing the prediction accuracy during the testing phase. The designs of the SCSQ, mask deformable attention, and SFN modules were explored for their usefulness in training the Chinese character stroke detection network, with a check mark (√) indicating whether the specific technique or module was used.

Table 4 shows the ablation results of various options of the proposed deformable attention module based on the mask mechanism. Using the mask mechanism to resample the offset of deformable attention effectively improved detection accuracy, with a 1.5% increase in the AP. Adding the simple conditional spatial query module further improved the AP value by 1.1%. Using the proposed SFN module in this paper to split channels further improved the AP by 1.2%. Overall, using both the simple conditional spatial query module and the SFN module improved the AP value in this experiment. It can be seen that when either the SCSQ module or the SFN module was added alone, the SFN module led to more improvements than the SCSQ module.

In this paper, a mask mechanism is used to filter the sampled reference points and resample the invalid reference points to reduce the randomness of reference points during the feature updating processes. This enables the model to converge faster compared to not using a mask mechanism. Therefore, better detection performance can be achieved by a model with a mask mechanism in the same cycle compared to a model without it. Moreover, a simple conditional spatial query strategy is used in this paper to separate the content query and spatial query, reducing the dependence of the prediction task on the content embedding and accelerating the convergence of our model compared to models that do not use a simple conditional spatial query. Finally, a channel-splitting FFN network is adopted in this paper as the prediction head. The classification and regression tasks can focus on their respective features and interconnections, thereby improving the model’s detection accuracy rate. However, since a mask prediction branch, as well as a resampling strategy, are introduced in this study, and a conditional spatial query strategy is used to compute the conditional spatial query vector, additional computations are needed, and the runtime of the model is longer compared to the original deformable DETR.

## 5. Conclusions

In this study, a deformable DETR method based on a mask mechanism with a simple conditional spatial query for detecting Chinese character strokes is proposed. This method is utilized to address the problem of random sampling in the deformable attention module in the original deformable DETR, to further accelerate convergence speed, and to improve accuracy. The mask mechanism designed in this study can be used to effectively reduce the uncertainty of deformable attention in sampling, thus reducing unnecessary computational costs. The simple conditional spatial query module is added to significantly improve the detection performance of the model with only a small increase in the number of parameters. Moreover, for the transformer task, the final query vector output of the decoder is split to specify the specific predictions for different tasks, which can be used to slightly improve the model’s performance without any increase in the computational cost and number of parameters.

This method provides a new solution for Chinese character stroke detection tasks with an improved detection paradigm. Moreover, as a method capable of handling Chinese character strokes, this method can accomplish the Chinese character writing task using robotic arms at the stroke level. Meanwhile, in addition to detecting strokes, a library for stroke order also needs to be built. The rules are established for each stroke of each Chinese character in order to complete the process of Chinese character reduction.

Although we have completed the task of detecting the strokes of standard Chinese characters, the task of detecting the strokes of handwritten Chinese characters is still difficult due to their irregularity and stylistic heterogeneity. In the future, we will focus on stroke detection and the restoration of handwritten Chinese characters. Meanwhile, this work has important implications for early education in Chinese character calligraphy, the dissemination of multi-font Chinese character graphics on social networks, and writing using industrial robotic arms.

## Figures and Tables

**Table 1:** The 52 stroke categories for standard Chinese character images based on Chinese Pinyin.

dian	fandian	duanheng	heng	changheng
shu	zuoxieshu	youxieshu	pie	shupie
fanpie	na	ti	piedian	shuti
hengzheti	wangou	shugou	shuwangou	xiegou
wogou	henggou	hengzhegou	hengzhexiegou	tizhegou
hengzhewangou	hengzuozhewangou	hengpiewangoungzhegou	hengpiewanwan	hengzhezhezhegou
hengzuozhezhezhegou	shuzhezhegou	shuwan	hengzhewan	hengzhe
hengzuozhe	xieshuzhe	shuzhe	shutizhe	piezhe
banpiezhe	hengpie	hengxiaopie	tixiaopie	banhengpie
hengna	hengzhezhepie	shuzhepie	hengxiegou	shuzhezhe
hengzhezhe	hengzhezhezhe			

**Table 2:** The comparative detection results of the deformable DETR and the SCSQ-MDD (simple conditional spatial query mask deformable DETR) on the test set. The metrics used to evaluate detection accuracy include the AP, AR, params, FLOPS, and FPS.

Method	Epochs	AP	${\mathrm{AP}}_{50}$	${\mathrm{AP}}_{75}$	${\mathrm{AP}}_{M}$	${\mathrm{AP}}_{L}$	AR	Params	FLOPS	FPS
Deformable DETR [35]	150	79.8	90.5	89.1	71.8	79.8	90.6	40M	144G	5
Mask Deformable DETR	150	81.3	92.5	91.7	71.7	81.3	91.4	41M	185G	4
SCSQ-MDD	150	83.6	93.6	93.0	71.7	81.7	91.7	46M	185G	4

**Table 3:** The comparative detection results of the SCSQ-MDD and mainstream methods on the test set. The metrics used to evaluate detection accuracy include the AP, ${\mathrm{AP}}_{50}$, ${\mathrm{AP}}_{75}$, and AR. A check mark (√) indicates whether to enable the SFN module.

Method	Backbone	SFN	AP	${\mathrm{AP}}_{50}$	${\mathrm{AP}}_{75}$	${\mathrm{AP}}_{M}$	${\mathrm{AP}}_{L}$	AR
Faster RCNN [8]	ResNet50		78.5	96.4	94.4	68.9	78.5	82.6
ATSS [37]	ResNet50		71.8	89.8	83.1	79.6	71.7	79.0
YOLOv5	ResNet50		84.1	94.1	–	–	–	–
YOLOv5	ResNet50	*√*	84.9	94.9	–	–	–	–
YOLOv7 [17]	ResNet50		87.9	95.0	–	–	–	–
SCSQ-MDD	ResNet50	*√*	88.1	95.6	95.5	72.1	88.2	92.9
SCSQ-MDD	ResNet101	*√*	88.6	96.9	96.0	75.1	88.6	93.5

**Table 4:** Ablation results of the SCSQ-MDD network structure on the test set. “Mask Deformable Attn.” refers to the deformable attention module based on the mask mechanism and “Simple Conditional Spatial Query (SCSQ)” refers to the simple conditional spatial query strategy. A check mark (√) indicates whether to enable the specified module.

Deformable DETR	Mask Deformable Attn	SCSQ	SFN	AP	>AP50	AP75	AR
*√*				79.8	90.5	89.1	90.6
*√*	*√*			81.3	92.5	91.7	91.4
*√*	*√*	*√*		82.4	92.4	92.0	91.3
*√*	*√*		*√*	82.5	93.1	92.6	90.7
*√*		*√*	*√*	81.5	91.1	89.7	92.1
*√*	*√*	*√*	*√*	83.6	93.6	93.3	91.7

## Data Availability

Data are contained within the article.
